# Experimental formation enthalpies for intermetallic phases and other inorganic compounds

**DOI:** 10.1038/sdata.2017.162

**Published:** 2017-10-24

**Authors:** George Kim, S. V. Meschel, Philip Nash, Wei Chen

**Affiliations:** 1 Illinois Institute of Technology, Department of Mechanical, Materials and Aerospace Engineering, Chicago, Illinois 60616, USA; 2 Illinois Institute of Technology, Thermal Processing Technology Center, Chicago, Illinois 60616, USA

**Keywords:** Thermodynamics, Materials science, Techniques and instrumentation

## Abstract

The standard enthalpy of formation of a compound is the energy associated with the reaction to form the compound from its component elements. The standard enthalpy of formation is a fundamental thermodynamic property that determines its phase stability, which can be coupled with other thermodynamic data to calculate phase diagrams. Calorimetry provides the only direct method by which the standard enthalpy of formation is experimentally measured. However, the measurement is often a time and energy intensive process. We present a dataset of enthalpies of formation measured by high-temperature calorimetry. The phases measured in this dataset include intermetallic compounds with transition metal and rare-earth elements, metal borides, metal carbides, and metallic silicides. These measurements were collected from over 50 years of calorimetric experiments. The dataset contains 1,276 entries on experimental enthalpy of formation values and structural information. Most of the entries are for binary compounds but ternary and quaternary compounds are being added as they become available. The dataset also contains predictions of enthalpy of formation from first-principles calculations for comparison.

## Background & Summary

The standard enthalpy of formation of a compound is the energy associated with the reaction to form the compound from its component elements (at the pressure of 1 atm. and the temperature of 298 K). The standard enthalpy of formation plays a pivotal role in the definition of Gibbs energy of formation of a compound^1,2^ and is widely used as input in thermodynamic models such as those used in the CALPHAD approach to calculate phase diagrams^3–10^. For example, Reichmann *et al.*^4^ measured standard enthalpies of formation of compounds in the Cd-Pr system and used the data to improve the agreement between the calculated Cd-Pr phase diagram and experimental results. New phases identified in calorimetry experiments can also help amend existing experimental phase diagrams. For instance, Bittner *et al.*^11^ discovered a new phase (Ge_4_Ti_5_, space group *Pnma*) while performing calorimetry experiments in the Ge-Ti system for which there was little literature prior to their work. In addition, enthalpy of formation data of known compounds are valuable resources for empirical prediction of phase stability of new phases in materials discovery. Recently, Miracle *et al.*^2^ estimated the Gibbs energies of formation of over 1,000 binary intermetallic compounds from their respective enthalpies of formation. Using this approximation, they predicted the stability region of novel high entropy alloys at 1,500 K^2^.

Calorimetry provides the only direct method by which the enthalpy of formation is experimentally measured. High-temperature calorimetric methods were used for the measurements of enthalpy of formation in the current dataset. The advantage of high-temperature calorimetry is that it enables the calorimetric study of compounds with high melting points^3^. However, conducting these experiments is a time-consuming process. For example, a high-temperature calorimetry experiment to determine the enthalpy of formation for just a single compound can take about 18 h. The determination of the enthalpy of formation involves the measurement of the heat of reaction to form the compound, the heat content of the compound itself, and the heat content of a standard reference material for calibration. All measurements are made with multiple samples to quantify the uncertainty of measurements. Due to the large amount of time and resources needed for these measurements, calorimetric measurement results are scarce and non-existent for many systems^12^. In this work, we present a large dataset of experimentally determined enthalpies of formation from our measurements and literature, including unpublished results obtained in our group at the Illinois Institute of Technology (IIT). Most of the entries are binary intermetallic compounds with transition elements, and rare-earth elements, but ternary and quaternary compounds are being added as they become available. The dataset can also be used to compare experimental measurements of enthalpies of formation of compounds in other critically assessed experimental databases such as the SGTE Substances Database, v3.3 which contains standard enthalpies of formation 3,064 condensed stoichiometric compound phases^13^.

The dataset presented is mostly based on the work of O.J. Kleppa’s group at the University of Chicago, and Philip Nash’s group at IIT. The dataset contains 1,276 entries on experimental enthalpy of formation values and structural information such as the space group and Pearson symbol. In the Methods section, the calorimeter setup, calorimetric methods (direct synthesis, solution, solute-solvent drop), and equations used to determine the enthalpies of formation are briefly described. The full dataset is in a JSON file, described in the Data Records section, accessible from the Figshare-repository (Data Citation 1) and will be updated regularly as more measurements are made. In the Technical Validation section a comparison of measurements made by different research groups is made to demonstrate the reproducibility of the experimental results. The dataset also has Density Functional Theory (DFT) predictions of enthalpies of formation from the Materials Project database^14^ and the Open Quantum Materials Database (OQMD)^15,16^ for comparison. The experimental measurements are of great values as benchmarks for first principles calculations.

## Methods

High-temperature calorimetric measurements were performed to collect the enthalpy of formation values in this dataset^1^. Three different high-temperature calorimetric methods are utilized in this dataset: direct synthesis, solution, and solute-solvent drop calorimetry. The developments in high-temperature calorimetry have enabled the study of compounds with high melting temperatures that require high temperatures to either make the component elements fully react and form the desired solid-state compound or melt and form a liquid phase. The choice between the three methods used in this work depends on experimental considerations. The direct synthesis method measures the enthalpy of formation directly, since the formation of the compound occurs within the calorimeter itself. Whereas in solution calorimetry and solute-solvent drop calorimetry, the compound is synthesized before the calorimetry measurements and the heat of dissolution of the compound is measured^3^. Solution or solute-solvent drop calorimetry is sometimes used instead of direct synthesis calorimetry because for some compounds, the temperature required for the component elements to react is too high or the reaction rate is too slow for the direct reaction method, resulting in residual unreacted component elements and inaccurate enthalpy of formation results^1^.

The functioning of a calorimeter relies on the precise measurement of temperature change, good insulation to avoid heat loss to the surroundings, and the presence of protective gas to avoid oxidation of the sample during the experiment^17^. The experimental setup and methods are described in further detail below, and the precision of the various methods is examined in the Technical Validation section.

### Experimental setup

The calorimetric measurements performed by the Kleppa and Nash groups used a Calvet type calorimeter designed and built by O.J. Kleppa. The design of the Kleppa calorimeter is described in ref. 1. The sample crucible is composed of boron nitride. In rare cases when the sample reacts with boron nitride, a beryllium oxide crucible is used. The sample section is further protected from oxidation by stainless steel foils and zirconium ‘gettering’ tubes above the crucible. All experiments are performed under argon gas which is purified by passing over titanium chips at 1,173 K. The experiments are carried out with the temperature in the calorimeter held at 1,373 K, except for some cases where the calorimeter temperature was lowered to 1,273 K due to experimental considerations such as the vapor pressure of a component element being too high at 1,373 K^18^. In cases where analysis of reacted samples show unreacted component elements, a commercial Setaram Ligne 96 drop calorimeter is used for measurement at a higher temperature to ensure the reaction goes to completion. The principle and design of both calorimeters is very similar with the thermopile and reference junction sections located vertically on a ceramic tube surrounding the sample reaction crucible.

The calorimeters have a thermopile embedded in alumina cylinders that surround the sample section and the temperature from the thermopile is recorded over time. The area under the temperature-time curve is proportional to the heat effect. The Kleppa calorimeter calibration is made using pure copper and this is performed once every two weeks. The Setaram calorimeter calibration measurements are performed after each sample drop using a NIST sapphire standard reference material (SRM 720). The latter calibration technique is now also being applied for some of the Kleppa calorimeter measurements.

### Direct synthesis

In the direct synthesis method, the component elements of the compound (in powder form) are mixed in the appropriate molar ratio, compressed into small pellets of about 2 mm in diameter and then dropped from room temperature into the calorimeter^3^. The high temperature inside the calorimeter enables the components to react completely and the heat of reaction is measured. This reaction is represented in equation (1) with component element *A* and component element *B* both in their standard states at 298 K reacting to form compound *A*_*α*_*B*_*β*_ at the reaction temperature *T.* The *s* represents the solid state, and *α* and *β* represent the mole fractions of the elements. Δ_*r*_*H*^*T*^ represents the measured heat of reaction at temperature *T*. Multiple pellets can be dropped into the calorimeter (one at a time and making sure that thermal equilibrium is regained before a consecutive drop). The multiple drops are used to calculate an average and s.d. of the measurement. The pellets which are now composed of the reaction product, compound *A*_*α*_*B*_*β*_ in equation (2), are removed from the calorimeter and the pellets are once again dropped from room temperature into the calorimeter, at the reaction temperature *T*, to measure the heat content of the compound. The calorimeter temperature for both measurements must be the same as well as the state of the samples at the calorimeter temperature. The heat content of the compound is represented by Δ*H*^*T−298*^(*A*_*α*_*B*_*β*_) in equation (2). Again, an average and s.d. is calculated from multiple sample measurements.

The reaction that corresponds to the standard enthalpy of formation of the compound *A*_*α*_*B*_*β*_ is obtained by subtracting equation (2) from equation (1), and is given in equation (3). In equation (3) the component elements *A* and *B* are both in their standard states at 298 K and they react to form the compound *A*_*α*_*B*_*β*_ in its standard state at 298 K. The standard enthalpy of formation Δ_*f*_*H*^*298*^(*A*_*α*_*B*_*β*_) is determined in equation (4) by subtracting the heat content of the compound, Δ*H*^*T−298*^(*A*_*α*_*B*_*β*_), from the heat of reaction, Δ_*r*_*H*^*T*^(*αA*+*βB*).

During the time between the heat of reaction and the heat content experiments, the samples are kept in a vacuum desiccator to prevent reaction with oxygen or moisture. The uncertainty in the standard enthalpy of formation is calculated by adding the s.d. of the heat content measurements, the s.d. of the heat of reaction measurements, and the s.d. of the calibration measurements in quadrature. The composition of the reacted compound is examined with energy dispersive spectroscopy (EDS) to check for unreacted or impurity phases. Then the sample is examined by x-ray powder diffraction analysis (XRD) to determine the structure of the phase or phases present^5^.

(1)αA(s,298K)+βB(s,298K)=AαBβ(s,T)ΔrHT(αA+βB)

(2)AαBβ(s,298K)=AαBβ(s,T)ΔHT−298(AαBβ)

(3)αA(s,298K)+βB(s,298K)=AαBβ(s,298K)ΔfH298(AαBβ)

(4)ΔfH298(AαBβ)=ΔrHT(αA+βB)−ΔHT−298(AαBβ)

### Solution calorimetry

The solution calorimetry method relies on rapid solution of both the components and the reacted products in the selected solvent. The solvent is usually a low melting metal such as tin, copper or aluminum^3^. It is important to check that the solute is completely dissolved. A bubbling tube can be utilized to help the sample dissolve. The heat of dissolution is measured for a mechanical mixture of the component elements. In equation (5), *A* and *B* represent the component elements that are dropped from 298 K into the solvent that is at temperature *T.* The heat of dissolution at temperature *T* of the component elements *A* and *B* is Δ_*soln*_*H*^*T*^(*αA*+*βB*). The heat of dissolution of the reacted compound is also measured. In equation (6) the compound *A*_*α*_*B*_*β*_ is dropped from 298 K into the solvent which is at the same temperature *T* as before. The heat of dissolution associated with this reaction is Δ_*soln*_*H*^*T*^(*A*_*α*_*B*_*β*_). The reaction that corresponds to the standard enthalpy of formation of the compound *A*_*α*_*B*_*β*_ is obtained by subtracting equation (6) from equation (5), and is given in equation (7). In equation (8) the standard enthalpy of formation, Δ_*f*_*H*^*298*^(*A*_*α*_*B*_*β*_), of the compound is determined by subtracting the heat of dissolution from equation (6) from the heat of dissolution from equation (5).

(5)αA(s,298K)+βB(s,298K)+solvent(l,T)=solution(l,T)ΔsolnHT(αA+βB)

(6)AαBβ(s,298K)+solvent(l,T)=solution(l,T)ΔsolnHT(AαBβ)

(7)αA(s,298K)+βB(s,298K)=AαBβ(s,298K)ΔfH298(AαBβ)

(8)ΔfH298(AαBβ)=ΔsolnHT(αA+βB)−ΔsolnHT(AαBβ)

The compound *A*_*α*_*B*_*β*_ in equation (6) should be examined by EDS and XRD to confirm there are no additional phases, and to determine its structure.

### Solute-solvent drop calorimetry

This method was developed in the Kleppa lab at the University of Chicago in 1984 for compounds which have high melting point that it is not possible to achieve complete reaction at 1,473 K by the direct synthesis method^19^. In this method, an unreacted mixture of the component elements is dropped into the calorimeter with a solid solvent material chosen such that the combination will form a liquid phase in the calorimeter. In a subsequent experiment the compound is dropped into the calorimeter with the same ratio of solvent material. As the liquid phase is formed the heat of dissolution is measured. As with solution calorimetry, the solvent material should not react with the components of the compound^9^. The formation enthalpy calculation is identical to that of the solution calorimetry method. The reacted compound should be examined by EDS and XRD to confirm there are no additional phases and to determine its structure.

## Data Records

The dataset of experimentally measured standard enthalpies of formation, crystal structure information and other information is reported in enthalpy_formation.json [Data Citation 1]. The full list of recorded attributes, referred to as keys in the JSON format, is described in Table 1. The dataset contains 1,276 entries and can be retrieved from the Figshare repository [Data Citation 1]. The key ‘notes’ contains text with comments about the experimental results. For example, some compounds in the dataset were revealed by XRD analysis to have multiple crystalline modifications which are listed. A small amount of second phase can be tolerated as it will not substantially affect the result and this is usually taken as 5% (volume fraction determined by quantitative XRD analysis). If up to 10% of a second phase is present then the entry has a comment in the notes key labeling the entry as an ‘Indicative Result’.

Another important key in Table 1 is the ‘uncertainty’ key. There are three types of measurement uncertainties recorded in the JSON file due to differing practices used by different sources. Some enthalpy of formation values were published with their measurement uncertainties as standard errors instead of s.d.’s. The s.d. describes how much individual data points differ from the sample mean. The standard error of the mean describes probabilistically how the mean varies given the current sample size. There is also the case where uncertainties are reported simply with an upper and lower limit of the error (i.e., ±0.01 eV/atom) without details on how they were calculated. In this case the uncertainty is simply labeled as ‘experimental_error’.

One of the ‘properties’ obtained from the DFT databases is the ‘Energy above convex hull’ property. If a structure has an energy above convex hull value of 0 the structure is the most thermodynamically stable one at its composition. If the value is positive the structure is predicted to decompose into other more thermodynamically stable compounds. As such it is a useful property to screen compounds based on thermodynamic stability. For example, Ni_2_CoGa was a compound that was erroneously reported to be thermodynamically stable^20^, but OQMD’s energy above hull value for the compound indicated that the structure was not stable. OQMD’s prediction was confirmed experimentally in an investigation by Yin, M., Nash, P., Chen, W. and Chen, S^21^. Additionally, experimentalists could use this calculated value to discover compounds with polymorph structures by examining structures with small positive values for the energy above the convex hull.

### File format

Each entry is a JSON object in the dataset and corresponds to a compound. The JSON format is based on a series of key-value pairs. The JSON keys and their descriptions are listed in Table 1.

## Technical Validation

### Experimental procedure to limit confounding variables

The experimental procedures described above ensure reproducible measurements by limiting confounding variables. The reaction of the component elements in the sample crucible occur under a protective atmosphere of Argon gas which is purified by passing it over titanium chips at 1,173 K. The procedure keeps reaction with oxygen to a minimum. The crucible is made of boron nitride and after every experiment the crucible is checked to make sure that it did not react with the sample. The formation enthalpy is measured through a two-step process. The first step measures the heat of reaction when the component elements react to form the compound of interest. The second step measures the heat content of the reacted compound. Between the two steps the samples are kept in a vacuum desiccator to prevent reaction with oxygen or moisture. This ensures that the reacted compound does not form oxides or undergo other side reactions that affect the measurement. Additionally, the weight of the samples is checked after each experiment to check for significant mass loss. Each reported enthalpy of formation is based on individual measurements of multiple samples (7 or more). The setup is such that multiple samples of the same compound can be measured in one setting; after each sample is dropped into the crucible through a drop tube in the calorimeter, sufficient time is passed for the calorimeter to reach thermal equilibrium. Additionally, after each sample is dropped in and measured, a reference standard is dropped in for a calibration measurement. The modified mean of the calibration measurements is calculated by calculating the average after removing the largest and smallest values. This calibration method is used for all Setaram calorimeter measurements and some Kleppa calorimeter measurements.

### Comparison of precision of calorimetric methods

**Figure 1:**
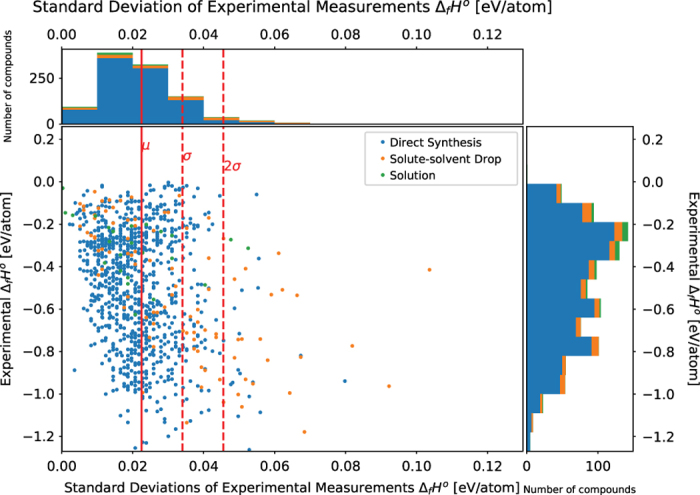
Representation of the uncertainty in experimental measurements. Plot of measurement uncertainties grouped by calorimetric method. The scatterplot x-axis is the measurement uncertainty of the experimental measurements of the standard enthalpy of formation. The y-axis is the experimentally measured standard enthalpy of formation. The solid red line is the mean of the measurement uncertainty (*μ*=0.0225 eV/atom), and the dashed red lines are one and two s.d.’s (*σ*=0.0115 eV/atom). Histograms on each axis indicate the number of measurements in each respective bin.

Figure 1 is a graphical representation of the uncertainty of the experimental results in the dataset, grouped by the calorimetric method used. The uncertainty is quantitatively described by the s.d. of the measured standard enthalpy of formation. The s.d. of the standard enthalpy of formation measurement is calculated by adding the s.d. of the heat of reaction measurements, the s.d. of the heat content measurements, and the s.d. of the calibration measurements in quadrature, using equation (9).

(9)σmeasurement=(σheatofreaction)2+(σheatcontent)2+(σcalibration)2

Where *σ*_*measurement*_ is the measurement s.d., *σ*_*heat of reaction*_ is the s.d. of heat of reaction measurements, *σ*_*heat content*_ is the s.d. of heat content measurements, and *σ*_*calibration*_ is the s.d. of calibration measurements. This method of computing propagated error assumes that the measured quantities are independent and that the measurement errors have a normal distribution. Instrumental errors such as a skewed distribution of measurements or drift in the instrument measurements are both mitigated by the experimental procedure. If the distribution of instrumental errors is skewed, taking the average measurement of the calibration tests will introduce a systematic error. If there is a drift in the instrumental measurements the error of the measurements get worse as the experiment progresses. The effect of a possible skew in the distribution in calibration measurements is mitigated by calculating a modified mean which is a more robust estimator of central tendency compared to the mean. The possible drift in measurements is mitigated by taking calibration measurements throughout the entire test, one after every sample. Figure 1 demonstrates the reproducibility of the experimental methods used. There are three different calorimetric methods: direct synthesis calorimetry, solution calorimetry, and the solute-solvent drop calorimetry. Figure 1 shows that the three methods are comparable in their measurement precision. 906 measurements by direct synthesis, 94 by solute-solvent drop, and 26 by solution calorimetry are plotted in Fig. 1. The dataset contains experimental results for measurements with the reaction temperature as the reference temperature instead of 298 K. These measurements were omitted in Fig. 1 for consistency. The solid red line represents the mean (*μ*) of the measurement uncertainty which was 0.0225 eV/atom. The dashed red lines represent one and two s.d.’s away from the mean (1*σ*, 2*σ*) respectively. The s.d. was 0.0115 eV/atom. Of the 906 direct synthesis measurements, 3.1% were more than 2 s.d.’s away from the mean. Of the 94 solute-solvent drop measurements, 30.8% were more than 2 s.d.’s away from the mean. In the solution calorimetry measurements, there was a single large outlier not plotted in Fig. 1 which was the measurement of Nd_2_Al using HCl acid as a solvent with a standard enthalpy of formation of −1.073 eV/atom and a s.d. of 0.243 eV/atom. Of the remaining 25 solution calorimetry measurements, 12.0% were more than 2 s.d.’s away from the mean. With a few exceptions, this demonstrates that the three calorimetry methods have comparable precision of measurements. It should be noted however, that the number of solute-solvent drop and solution calorimetry measurements are much smaller than the number of direct synthesis calorimetry measurements, meaning that the description of the spread of the measurements of solute-solvent drop and solution calorimetry may not be as accurate as compared to that of direct synthesis calorimetry. It should also be noted that the vertical patterns visible in Fig. 1 is an artifact of the number of significant digits used in reporting the s.d.. Most of the literature published reported their uncertainty with a precision of 1 meV/atom.

### Validation through comparison of experimental measurements by different research groups

To demonstrate that the experimental results are reproducible and that measurements made by different research groups are comparable we list in Table 2 calorimetry results of identical compounds measured by different research groups. The number of comparable replication measurements by different research groups in the dataset is small, and Table 2 lists those that are comparable. Of the 76 compounds that have multiple entries, 31 are not comparable because some research groups reported enthalpies of formation with respect to temperatures other than 298 K. There are also 28 compounds that have replication measurements that are comparable, but are reported by members of the same research group, and therefore are not included in Table 2. For calorimetry experiments, it is convention to report enthalpies of formation in units of kJ/mol of atoms and for DFT calculations it is convention to report enthalpies of formation in eV/atom. The dataset has values in both units. For Table 2 the original units of kJ/mol of atoms were converted to eV/atom to follow the rest of the text. 1 kJ/mol of atoms is 0.0103 eV/atom. There are 17 compounds in Table 2, with two measurements for each reported by different sources. The phase, structure, enthalpy of formation (s.d.), and absolute difference is listed. For all the experimental results in Table 2 the absolute difference in experimental measurements has the same order of magnitude or less than the uncertainty of the measurements. This indicates that experimental measurements made by independent research groups are comparable to each other, however, it is noted that the number of comparable replication measurements between different research groups is small and more results published by other research groups could be added.

### Comparison of DFT and experimental results

**Figure 2:**
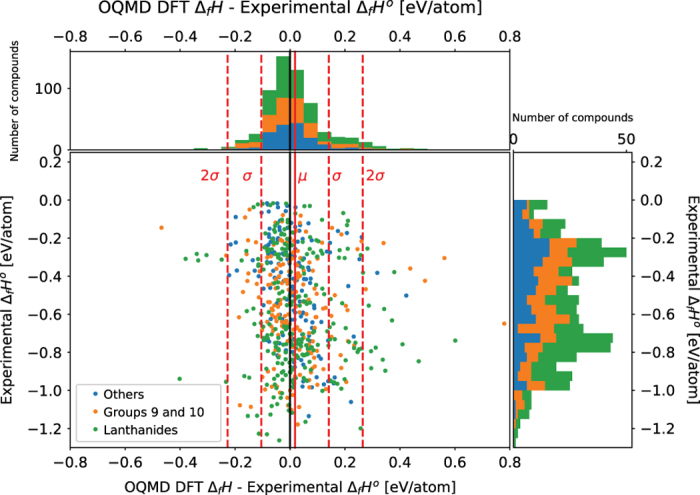
Comparison between Experimentally Measured Standard Enthalpy of Formation with calculated Enthalpy of Formation values from OQMD. The x-axis of the scatter plot is the difference between the calculated value and the experimentally measured value. The y-axis is the experimentally measured standard enthalpy of formation. The solid red line represents the average of the difference in values, which is 0.0183 eV/atom. The dashed red lines represent one and two s.d.’s away from the mean. The s.d. is 0.123 eV/atom. There are 607 points in total, and 31 points that are more than 2 s.d.’s away from the mean. The most common features shared by these outlier compounds is that they either contain Lanthanide elements or elements from groups 9 and 10 on the periodic table.

**Figure 3:**
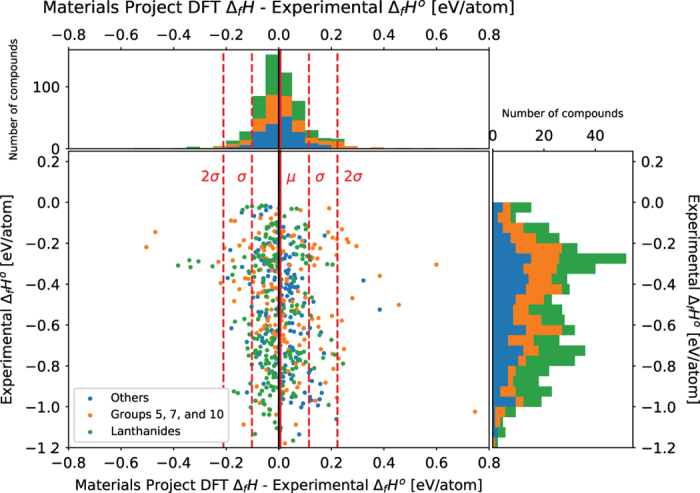
Comparison between Experimentally Measured Standard Enthalpy of Formation with calculated Enthalpy of Formation values from Materials Project. The x-axis of the scatter plot is the difference between the calculated value and the experimentally measured value. The y-axis is the experimentally measured standard enthalpy of formation. The solid red line represents the average of the difference in values, which is 0.00616 eV/atom. The dashed red lines represent one and two s.d.’s away from the mean. The s.d. is 0.108 eV/atom. There are 562 points in total, and 25 points that are more than 2 s.d.’s away from the mean. Most of these outliers contain either Lanthanide elements or elements from groups 5, 7, and 10 on the periodic table.

In addition to a comparison between replication experiments, a comparison between experimental measurements and first-principles calculations based on density functional theory is also made. Figures 2 and 3 compares the experimentally measured standard enthalpy of formation values with the calculated values on OQMD^15,16^ and Materials Project^14^ respectively. In Figs 2 and 3 The x-axis of the scatter plot is the difference between the calculated value and the experimentally measured value. The y-axis is the experimentally measured standard enthalpy of formation. The DFT calculated values are with respect to 0 K whereas the standard enthalpy of formation is with respect to 298 K. The solid red line represents the average of the difference in values. The dashed red lines represent one and two s.d.’s away from the mean. The compositions of the compounds and the positions of the plot points were examined for any patterns and clusters. Also, compounds in the dataset for which the reference temperature of the reported enthalpy of formation that is not 298 K are omitted from the plots.

Figure 2 compares the experimental results with DFT predictions on OQMD. The average difference in values between OQMD and the experimental measurements is 0.0183 eV/atom and the s.d. is 0.123 eV/atom. There are 607 compounds compared. Not all compounds in the dataset were found on OQMD. There are 31 outliers that are 2 s.d.’s away from the mean. The most common features shared by the outlier compounds is that they contain lanthanide elements, or elements from groups 9 and 10 in the periodic table (with two exceptions MnSnAu, and FeGe_2_). In the case of the half-Heusler compound MnSnAu (space group F-43m), which does not contain a lanthanide, group 9, or group 10 element, there are three inequivalent atomic arrangements possible^22^ and it may be the case that the experimentally measured structure and the OQMD structure have inequivalent atomic arrangements. There is only one OQMD entry for MnSnAu with a calculated enthalpy of formation. The specific atomic arrangements of the half-Heusler compounds were not reported for the experimental measurements. Table 3 lists the top 10 outliers ranked by absolute difference between the experimental and OQMD values.

Similar comparisons between calculated values from Materials Project and experimental measurements are plotted in Fig. 3. The average of the difference between the experimental measurements and the Materials Project values is 0.0062 eV/atom. The s.d. of the difference is 0.108 eV/atom. There are 562 compounds plotted in total and 25 compounds that are beyond 2 s.d.’s from the mean. The most common features shared within the outlier compounds is that they contain lanthanide series elements, or elements from groups 5, 7 and 10 on the periodic table (with two exceptions Zr_5_Pb_3_ and FeGe_2_). This is a similar situation with the comparison with the OQMD values where there is some disagreement between experimental measurements and DFT calculated values for intermetallic compounds with lanthanides. Table 4 below lists the 10 compounds with the largest absolute difference between the Materials Project DFT calculated values and the experimentally measured values.

In the case of the intermetallic compounds with lanthanide elements the disagreement between the experimental measurements and DFT may be due to the large electronic correlation effects of the 4f-electrons^23^. We should note that DFT predictions of enthalpies of formation are with respect to a reference temperature of 0 K, and that some of the differences with experimental measurements can be attributed to enthalpic and entropic contributions at finite temperatures^16^. The OQMD database and Materials Project database reduced the systematic error introduced by elements with phase transformations between 0 and 298 K. In the submitted experimental dataset, the constituent elements with low temperature phase transformations are Ce, Li, Ti and Sn. The OQMD database calculated the difference between the energies of the ground state and the room-temperature structures. Sn had the largest difference which was 42 meV/atom which has the same order of magnitude as the average experimental uncertainty. The systematic error was reduced by making corrections to the chemical potentials of the elements in the calculation of the enthalpy of formation. The magnitude of the corrections was calculated by solving the chemical potentials using a least squares fitting method using experimental formation enthalpies of compounds containing these constituent elements and the DFT calculated total energies of the compounds^16^. In comparisons between the experimental results of compounds containing constituent elements with low temperature phase transformations the calculated enthalpies of formation only have an average absolute error of about 0.1 eV/atom in both the Materials Project database and the OQMD database. The average uncertainty of the experimental standard enthalpy of formation measurements are one order of magnitude lower.

Of the 607 compounds plotted in Fig. 2, 264 belong to the category of compounds containing lanthanide series elements, 180 belong to the category of compounds containing elements from groups 9 and 10, and 163 belong to the category labelled ‘Others’. Of the 31 outlier compounds, only 1 compound FeGe_2_ belongs to the ‘Other’ category. Therefore, containing a lanthanide series element or a group 9 or 10 element is a better predictor for whether a compound might be an outlier.

In Fig. 3, of the 562 compounds plotted in Fig. 3, 215 belong to the category of compounds containing lanthanide series elements, 173 belong to the category of compounds containing elements from groups 5, 7, or 10, and 174 belong to the category labelled ‘Others’. Of the 25 outlier compounds only 2 compounds, Zr_5_Pb_3_ and FeGe_2_ belongs to the ‘Other’ category. Again, containing a lanthanide series element or an element from groups 5, 7, and 10 is a better predictor for whether a compound might be an outlier. Other confounding variables that apply more generally and can also contribute to the difference may be human transcription errors, an inaccurate ICSD entry for the crystal structure, or convergence to a magnetic configuration that is not the ground state magnetic configuration.

## Usage Notes

The dataset presented in this work provides researchers with 1,276 experimentally measured enthalpies of formation of intermetallic compounds with transition elements and rare-earth elements. The dataset also includes metal borides, metal carbides, and metallic silicides. The standard enthalpy of formation of a compound is a fundamental thermodynamic property that correlates with the phase stability and may be used with other thermodynamic data to calculate phase diagrams. In the design of alloys, calculated phase diagrams are useful for guiding areas of research and the availability of experimental thermodynamic data can help optimize calculated phase diagrams. Additionally, this dataset can be used to study trends in the enthalpy of formation, make comparisons with DFT measurements, and make comparisons with other experimental measurements. The precision and reproducibility of the measurements was demonstrated and so we expect the dataset to be a good baseline for comparisons with future DFT calculations and experimental measurements. Most of the entries in the dataset are binary compounds but ternary and quaternary compounds are being added as they become available.

## Additional Information

**How to cite this article:** Kim, G. *et al.* Experimental formation enthalpies for intermetallic phases and other inorganic compounds. *Sci. Data* 4:170162 doi: 10.1038/sdata.2017.162 (2017).

**Publisher’s note:** Springer Nature remains neutral with regard to jurisdictional claims in published maps and institutional affiliations.

## Supplementary Material



## Figures and Tables

**Table 1:** Description of the key-value pairs contained in the JSON file.

**Key**	**Value Datatype**	**Description**
id	String	Unique ID number for entries in dataset
composition	JSON object	key-value pairs of the component element’s chemical symbol (as a string) and molar fraction (as a float). {‘Ag’: 0.66, ‘Sc’: 0.33}
formula	String	Chemical formula of the compound
space_group	String	Space group in Hermann-Mauguin notation without spaces
pearson_symbol	String	Pearson symbol
standard_enthalpy_formation	Array	An array with two nested arrays. The first with the value in units of kJ/mole of atoms, and a string ‘kJ/mole of atoms’. The second with the value in units of eV/atom, and a string ‘eV/atom’
enthalpy_formation	Array	The enthalpy of formation measured with respect to reaction temperature and not 298 K. An array with two nested arrays. The first with the value in units of kJ/mole of atoms, and a string ‘kJ/mole of atoms’. The second with the value in units of eV/atom, and a string ‘eV/atom’
ref_temp	Int	The reference temperature in Kelvin. Standard enthalpy of formation has a reference temperature of 298 K
uncertainty	JSON object with Keys: ‘standard_deviation’, ‘standard_error’, ‘experimental_error’	The value assigned to the key is an array with two nested arrays. The first with the uncertainty in units of kJ/mole of atoms, and a string ‘kJ/mole of atoms’. The second with uncertainty in units of eV/atom, and a string ‘eV/atom’
notes	String	Contains a note, either says Indicative Result or Crystallographic Modifications
citation	String	The full reference to the article the measurement was published in
calorimetry_method	String	Calorimetric method, either Direct Synthesis, Solute-Solvent Drop or Solution
cas_reg_no	String	Chemical Abstracts Service unique identifier
mp_id	String	Materials Project ID
mp_formation_energy	Array	Enthalpy of formation from the Materials Project database. An array with two nested arrays. The first with the value in units of kJ/mole of atoms, and a string ‘kJ/mole of atoms’. The second with the value in units of eV/atom, and a string ‘eV/atom’
mp_e_above_hull	Array	Energy above convex hull from the Materials Project database. An array with two nested arrays. The first with the value in units of kJ/mole of atoms, and a string ‘kJ/mole of atoms’. The second with the value in units of eV/atom, and a string ‘eV/atom’
oqmd_id	String	OQMD ID
oqmd_formation_energy	Array	Enthalpy of formation from the OQMD database. An array with two nested arrays. The first with the value in units of kJ/mole of atoms, and a string ‘kJ/mole of atoms’. The second with the value in units of eV/atom, and a string ‘eV/atom’
oqmd_e_above_hull	Array	Energy above convex hull from the OQMD database. An array with two nested arrays. The first with the value in units of kJ/mole of atoms, and a string ‘kJ/mole of atoms’. The second with the value in units of eV/atom, and a string ‘eV/atom’
The details of the measurement of the enthalpy of formation for each entry in the JSON file are contained in key-value pairs. The table lists the keys, and a description of the value associated with each key.		

**Table 2:** Comparison of standard enthalpies of formation between different experimental groups.

**Phase (structure)**	**Standard enthalpy of formation (s.d.) (eV/atom)**	**Standard enthalpy of formation (s.d.) (eV/atom)**	**Absolute difference (eV/atom)**
Fe_2_NiAl (Pm-3m)	−0.289 (0.015)^19^	−0.320 (0.025)^20^	0.031
FeAl (Pm-3m)	−0.275 (0.011)^19^	−0.244 (0.017)^21^	0.031
NiAl (Pm-3m)	−0.641 (0.012)^22^	−0.604 (0.011)^21^	0.036
YMn_2_ (Fd-3m)	−0.029 (0.029)^23^	−0.025 (0.026)^24^	0.004
ZrPt (Cmcm)	−1.079 (0.019)^25^	−0.933 (0.104)^26^	0.146
ZrIr (Pm-3m)	−0.888 (0.04)^25^	−0.840 (0.021)^26^	0.049
ZrNi (Cmcm)	−0.523 (0.016)^27^	−0.534 (0.021)^26^	0.010
GdNi (Cmcm)	−0.310 (0.011)^27^	−0.267 (0.019)^28^	0.042
DyNi (Pnma)	−0.365 (0.016)^27^	−0.346 (0.020)^28^	0.019
CoTi (Pm-3m)	−0.428 (0.009)^27^	−0.459 (0.005)^26^	0.031
NiY (Pnma)	−0.341 (0.018)^12^	−0.379 (0.020)^29^	0.038
NiTi (Pm-3m)	−0.343 (0.011)^27^	−0.352 (0.021)^26^	0.009
TiPd (Pm-3m)	−0.535 (0.026)^27^	−0.550 (0.031)^26^	0.016
HfPd (Pm-3m)	−0.720 (0.018)^30^	−0.699 (0.040)^31^	0.022
LuPd (Pm-3m)	−0.944 (0.054)^32^	−0.985 (0.015)^27^	0.040
PdZr (Cmcm)	−0.635 (0.036)^32^	−0.685 (0.011)^27^	0.050
NiB (Cmcm)	−0.208 (0.020)^33^	−0.215 (0.011)^34^	0.006
Measurements of standard enthalpies of formation (and the s.d. of the measurements) of compounds performed by different research groups. There are 17 compounds with two measurements each for a total of 34 measurements being compared. Results were reported in units of kJ/mol of atoms but were converted to eV/atom to follow the rest of the text. 1 kJ/mol of atoms is 0.0103 eV/atom. The average of the absolute difference in measurements is 0.034 ev/atom of atoms. For all the results in the table the absolute difference has the same order of magnitude or lower than the measurement uncertainty.			

**Table 3:** Ten compounds with the largest absolute difference between the OQMD DFT calculated values and the experimentally measured standard enthalpy of formation values.

**Compound**	**Spacegroup**	**Standard enthalpy of formation (eV/atom)**	**Absolute difference (eV/atom)**
HfNiSn	F-43m	−0.649	0.78
Tb_5_Pb_3_	P63/mcm	−0.738	0.601
MnSnIr	F-43m	−0.305	0.562
MnGaIr	F-43m	−0.424	0.491
Tb_5_Ge_3_	P63/mcm	−0.847	0.468
TaRh_3_	Pm-3m	−0.145	0.468
PtPb	P63/mmc	−0.36	0.438
MnSnAu	F-43m	−0.502	0.423
Tb_5_Sn_3_	P63/mcm	−0.758	0.409
LuPt	Pnma	−0.939	0.402
The compound formula, space group structure, experimentally measured standard enthalpy of formation in eV/atom and the absolute difference between OQMD DFT and experimentally measured values are listed. The largest difference is 0.780 eV/atom.			

**Table 4:** Ten compounds with the largest absolute difference between the materials project DFT calculated values and the experimentally measured standard enthalpy of formation values.

**Compound**	**Space group**	**Standard enthalpy of formation (eV/atom)**	**Absolute difference (eV/ atom)**
HfSnPt	F-43m	−1.023	0.746
MnSnIr	F-43m	−0.304	0.599
TaPt_3_	P121/m1	−0.218	0.503
TaRh_3_	Pm-3m	−0.145	0.469
MnSnAu	F-43m	−0.501	0.456
Zr_5_Pb_3_	P63/mcm	−0.524	0.383
PtPb	P63/mmc	−0.359	0.383
LuB_2_	P6/mmm	−0.308	0.383
ErB_2_	P6/mmm	−0.282	0.34
TmB_2_	P6/mmm	−0.317	0.335
The compound formula, space group structure, standard enthalpy of formation in eV/atom and the absolute difference between Materials Project DFT and experimentally measured values are listed. The largest difference is 0.746 eV/atom.			
